# Assessment of knowledge and practices towards the diagnosis of arboviral infections amongst healthcare workers in Lagos State and the Federal Capital Territory: a cross-sectional study

**DOI:** 10.11604/pamj.2024.49.124.44504

**Published:** 2024-12-17

**Authors:** Abiodun Feyikemi Ipadeola, Olayemi Oluseun Akinnola, Olatunji Matthew Kolawole, Elvis Efe Isere, Timothy Adejoh Attah, Lazarus Onyema Omenyi, Odunola Dorcas Oladokun, Kafayat Olabisi Oyewunmi, Oyeronke Olufemi Ekun, Stellamaris Uche Oparaocha, Dorcas Yetunde Obazee, Comfort Ndaks, Egwu Emmanuel Ewa, Grace Iyabo Olasehinde

**Affiliations:** 1 Covenant University, Otta, Ogun State, Nigeria,; 2 Datametrics Associates Limited, FCT-Abuja, Nigeria,; 3 University of Ilorin, Kwara State, Ilorin, Nigeria,; 4 University of Ibadan, Oyo State, Ibadan, Nigeria,; 5 Lagos State University Teaching Hospital, Lagos State, Lagos, Nigeria,; 6 Randle General Hospital, Lagos State, Lagos, Nigeria,; 7 Orile Agege General Hospital, Lagos State, Lagos, Nigeria,; 8 Kubwa General Hospital, FCT-Abuja, Nigeria,; 9 Asokoro General Hospital, FCT-Abuja, Nigeria,; 10 Nyanya General Hospital, FCT-Abuja, Nigeria

**Keywords:** Arboviral infection, diagnostic practices, healthcare workers

## Abstract

**Introduction:**

in Nigeria, misdiagnosis of arboviral infections poses a significant public health threat to prompt diagnosis and optimum treatment. This study investigates the knowledge about arboviral diseases and diagnostic practices among healthcare workers (HCW) in Nigeria.

**Methods:**

a cross-sectional study with multistage sampling was carried out among healthcare workers in Lagos and federal capital territory (FCT). Semi-structured questionnaires were administered to healthcare workers. Descriptive and inferential statistics were performed using a 5% level of significance.

**Results:**

a total of 395 HCWs were enrolled from Lagos State (48.4%) and from the FCT (51.6%). Majority of the HCWs in Lagos State (49.2%) and the FCT (50.5%) were ≥35 years (P<0.001). In Lagos State, laboratory scientists were most represented (40.3%) compared to 40.7% of the nurses in the FCT (P=0.013). All participant in the FCT practiced in secondary facilities compared to Lagos State (82.2%) and 38.7% had <5 years´ experience compared to 51.3% of HCWs in Lagos State with >10 years´ experience (P<0.001). A higher proportion of HCWs in Lagos State showed greater awareness about arboviral infections (33.5%) compared to those in the FCT (6.4%) (p < 0.001). In Lagos State, male, age ≥35 years, having good knowledge, and prior training on arboviral diseases and their diagnosis, increased the odds of diagnosing an arboviral infection. Also, medical doctors as well as laboratory scientists were more likely than nurses to diagnose an arboviral infection (P<0.05). In the FCT, good knowledge and training on arboviruses and arboviral infection diagnosis increased the odds of arboviral disease diagnosis (P<0.05).

**Conclusion:**

our study found differences in arboviral infection diagnostic practices in Lagos and FCT. Enhancing training and dissemination of knowledge gained from training is vital to improve diagnosis and surveillance of arboviral infection and diseases.

## Introduction

Arboviruses are a diverse group of viruses transmitted primarily by arthropods such as mosquitoes and ticks and pose significant challenges to public health globally [1-9]. While this group of diseases has been identified in several countries across the globe, Nigeria´s varied climate, ranging from humid rainforests to arid savannas, provides ideal breeding grounds for vectors such as *Aedes* mosquitoes, which are responsible for transmitting arboviruses [1,10-13]. Additionally, rapid urbanization, population growth, and inadequate vector control measures further exacerbate the risk of arbovirus transmission, particularly in densely populated urban centers such as Lagos and the Federal Capital Territory (FCT) [1,11,13].

Nigeria has been significantly impacted by numerous infectious disease burdens, including those caused by arboviruses [1,13,14]. Despite the significant public health threat posed by arboviral infections in Nigeria, there remains a lack of awareness among many healthcare providers regarding these viruses, especially across several regions/States in Nigeria [11,13]. This could be attributed to the fact that are often nonspecific, leading to potential misdiagnosis with other common illnesses such as malaria, typhoid, and bacterial meningitis [11,15]. Additionally, previous studies have reported that the diagnosis of arboviral infections is not routinely included in laboratory investigations of samples from febrile patients at various hospitals in Nigeria [10,11,16]. As a result, cases of arboviral infections may go undetected, contributing to deficiencies in arbovirus surveillance and response, resulting in the poor identification and monitoring of arboviral disease outbreaks and increased risk of morbidity and mortality due to these diseases [10,11].

Additionally, States like Lagos and the Federal Capital Territory (FCT) experience high urbanization and enormous international presence, which increases the likelihood of cross-border disease entry into the country [17,18]. As a result, these States are considered epidemiologically relevant. While there is an urgent need for robust surveillance and control measures for arboviral diseases in Nigeria, there is limited knowledge about the level of awareness and diagnostic practices among healthcare workers (HCW) across different regions of Nigeria [11,16]. One study conducted in Sokoto State found that about 95% of the HCWs in a tertiary health facility had good awareness about Dengue fever [19]. Another study in south-eastern Nigeria however found that 64% of the HCWs had poor awareness about Zika virus [20]. Although no studies have been conducted specifically to assess the regional disparities, these studies suggest the potential for a regional disparity in the knowledge about arboviral infections across different States.

Given the diverse geographic and socioeconomic landscape, it is crucial to understand how certain factors can influence arbovirus diagnosis and detection practices among HCWs [1,11]. Regional disparities in healthcare infrastructure, access to diagnostic tools, training programs, and knowledge gaps could significantly impact HCWs´ ability to promptly diagnose and manage arboviral infections [11]. Thus, studies must be conducted to assess regional variations in knowledge and practices towards arboviral infections in Nigeria. Therefore, this study aimed to assess the disparities in the knowledge and practices of HCWs toward the diagnosis of arboviral infections in Lagos and FCT, Nigeria.

## Methods

**Study design:** this cross-sectional study was carried out on HCWs in Lagos and the FCT between October 2022 and September 2023.

**Figure 1:**
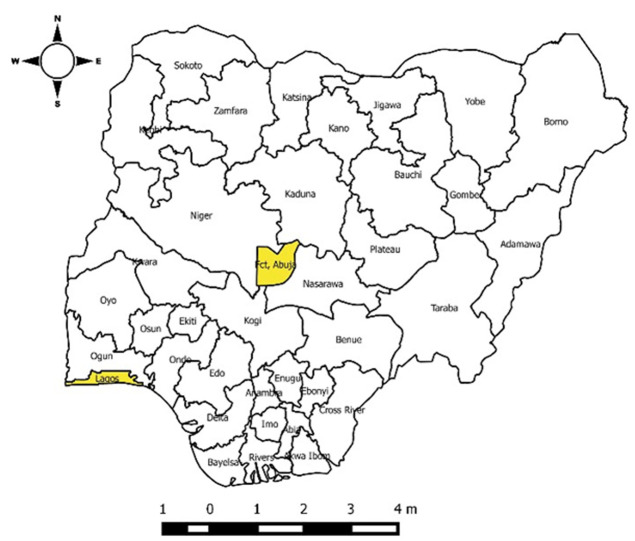
map of Nigeria showing the study locations

**Study area:** Nigeria is the most populous country in Africa, with an estimated population of over 200 million [21] and has six regional zones with varying ecologies, climates, and population characteristics. The 36 States and the federal capital territory are divided into these zones and are further divided into 774 LGAs or districts, and, 8812 administrative wards [22] (Figure 1).

**Eligibility criteria:** the study was conducted among three cadres of HCWs. This includes medical doctors, nurses, and laboratory scientists who are 18 years of age and above practicing in the selected facilities. Only consenting HCWs were interviewed for this study.

**Study sample:** a multistage sampling technique was employed in this study. In stage 1, we divided the 36 States and the FCT-Abuja into six categories according to the regional zones (Northeast region, Northwest region, North Central region, Southeast region, Southwest region, and South-south region), from which two regions; Southwest region and North Central region were randomly selected using a simple random technique (balloting). In stage 2, we listed the States in each selected region (6 in the Southwest Region and 6 States and the FCT-Abuja in the Northcentral region) from which Lagos State (Southwest) and FCT-Abuja (Northcentral) were selected purposively using the following criteria: rapid urbanization activities, high population density and high risk of arboviral disease importation from travels (international and local). In stage 3, a list of all secondary and tertiary health facilities in each State was obtained from the Ministry of Health authorities, and the participants were recruited. In each State, a minimum of secondary health facilities was selected for the study. Health facilities were purposively selected from each State based on patient volume and interest in collaboration. In Lagos State, Lagos State University Teaching Hospital (LASUTH); General Hospital, Orile-Agege & Randle General Hospital, Surulerewere selected, while in FCT-Abuja, Bwari General Hospital, Bwari; Kubwa General Hospital, Kubwa; Nyanya General Hospital, Nyanya and Asokoro General Hospital, Asokoro were selected for the study. Four facilities were selected in FCT-Abuja to compensate for the non-inclusion of a tertiary facility. A purposive sampling technique targeting all HCWs involved in the management of fever cases presented at the selected healthcare facilities was adopted to select eligible participants.

**Study instrument:** a semi-structured questionnaire was used to determine the knowledge and practice of HCWs towards arboviral infections. The questionnaire was divided into three sections. The first section of the questionnaire captured information on socio-demographic variables such as gender, age, cadre of HCW, facility level and years of experience. The second section contain questions assessing the level of awareness of arboviral infections and diagnosis among HCWs. For the knowledge score, respondents were scored using a 15-item scale. A point was assigned to the correct response to each question thereafter. The total score of each participant was divided by the total expected score (15 points) and multiplied by 100 to derive the percentage score. Respondents with scores of 50% and below were categorized as those with poor knowledge, while those with a score above 50% were categorized as those with good knowledge. The third section assesses the ability of HCWs towards arboviral infection diagnosis. This section assesses suspicion and diagnosis of arbovirus disease by HCWs.

**Data collection:** to ensure consistency in the way the questionnaires were administered, research assistants with field experience in data collection were recruited, trained, and deployed for data collection. Field supervisors were also recruited, trained, and deployed to monitor the data collection process. Responses from questionnaires were transcribed into Microsoft Excel 365 for data cleaning and coding.

**Study variables:** independent variables for this study include sociodemographic characteristics (sex, age, profession, facility type, and years of experience) and knowledge about arboviral infection while a dependent variable was defined by if the HCW had previously diagnosed an arboviral infection.

**Data management and analysis:** data analysis was performed using Stata (version 17). Socio-demographic characteristics and responses to questions on knowledge and practices towards arboviral infections and diagnosis were presented in frequency and percentages. Chi-squared analysis was performed to determine the socio-demographic factors associated with the study outcome, and this was stratified by State (Lagos and FCT). Fisher´s exact tests were applied when conditions for chi-square tests was not met. The associated p values were reported to aid in the interpretation of the results [23,24]. Only variables in the bivariate test at p-value of <0.2 were included in the multivariate model. Multivariate logistic regression analysis was used to determine the strength of the association. The analysis was performed using a 95% confidence level and 5% level of significance.

**Ethical approval:** ethical approval was obtained from the Covenant University Health Research Ethics Committee, the Lagos State Research Ethics Committee at the Lagos State University Teaching Hospital (LASUTH) with protocol approval number; LREC/06/10/1815, and the FCT-Abuja Health Research Ethics Committee with protocol approval number: FHREC/2022/01/174/05-09-22. Participants for the study were formally recruited and written informed consent was obtained from the respondents. They were made to understand that participation is voluntary and there was no consequence for non-participation. All information obtained from participants was de-identified and archived in a secured database only accessible to the authors to protect the confidentiality of study participants.

## Results

**Descriptive statistics of healthcare workers in Lagos and the FCT:** a total of 431 HCWs participated in this study, of which the females were most represented in the FCT (63.7%) and Lagos State (56.0%) (P: 118). 50.5% (103) of the HCWs in the FCT and 49.2% (94) in Lagos were 35+ years old. However, the least represented age group of HCWs in FCT was <24 years (12.3%) of age while 20.9% (40) of the HCWs in Lagos were within the 25-34 years age group respectively (P<0.001). A higher proportion of the HCWs were laboratory scientists in Lagos (40.3%) and nurses in the FCT (40.7%) while nurses in Lagos (26.7%) and medical doctors in the FCT (25.5%) were least represented (P: 0013). All HCWs in the FCT practiced in secondary health facilities compared to 82.2% (157) of the HCWs in Lagos that practiced in secondary facilities (p<001). Majority of the HCWs in Lagos had >10 years working experience, compared to 38.7% (79) of HCWs in the FCT with <5 years´ experience (P<0.001) (Table 1).

**Table 1:** sociodemographic characteristics of the respondents

Variables	Lagos (%)	FCT (%)	Total (%)	P value
Sex				0.118
Male	84 (44.0)	74 (36.3)	158 (40.0)	
Female	107 (56.0)	130 (63.7)	237 (60.0)	
Age				<0.001
Less than 24	57 (29.8)	25 (12.3)	82 (20.8)	
25 to 34	40 (20.9)	76 (37.3)	116 (29.4)	
35 and above	94 (49.2)	103 (50.5)	197 (49.9)	
Profession				0.013
Medical doctors	63 (33.0)	52 (25.5)	115 (29.1)	
Nurses	51 (26.7)	83 (40.7)	134 (33.9)	
Lab scientist	77 (40.3)	69 (33.8)	146 (37.0)	
Facility level				<0.001
Secondary	157 (82.2)	204 (100)	361 (91.4)	
Tertiary	34 (17.8)	0 (0.0)	34 (8.6)	
Year of experience				<0.001
Less than 5	42 (22.0)	79 (38.7)	121 (30.6)	
6 to 10 years	51 (26.7)	63 (30.9)	114 (28.9)	
More than 10 years	98 (51.3)	62 (30.4)	160 (40.5)	

Note: %=percent; lab=laboratory; FCT: Federal Capital Territory

**Knowledge about arboviral infections amongst healthcare workers in Lagos and the FCT:** a higher proportion (82.7%) of HCWs in Lagos had previous knowledge about arboviral infection compared to their counterparts in the FCT (33.8%) (P<0.001). While >95% of the HCWs in both States were unable to mention four to seven arboviral infections, 80.1% (153) in Lagos were able to mention one to three infections, compared to only 29.4% (60) in the FCT (P<0.001). Over 90% of the HCWs in both States were unable to mention four to six arboviral infections, while 66.2% (135) of the HCWs in FCT were unable to mention a single symptom compared to 17.3% (33) in Lagos (P<0.001). Compared to the FCT (6.4%), 33.5% (64) of the HCWs in Lagos had good knowledge about arboviral infection. However, this variation was insignificant (P: 102) (Table 2).

**Table 2:** level of knowledge about arboviral infection and diagnosis among healthcare workers in Lagos and the FCT

Variables	Lagos (%)	FCT (%)	Total (%)	p-value†
Heard of arboviral infection before				<0.001
Yes	158 (82.7)	69 (33.8)	227 (57.5)	
No	33 (17.3)	135 (66.2)	168 (42.5)	
Mention seven types of arboviral infections				<0.001
Participants who mentioned four to seven arboviral infections	5 (2.6)	9 (4.4)	14 (3.5)	
Participants who mentioned one to three arboviral infections	153 (80.1)	60 (29.4)	213 (53.9)	
Participants who mentioned none	33 (17.3)	135 (66.2)	168 (42.5)	
Mention six common symptoms of arboviral infections				<0.001
Participants who mentioned four to six symptoms of arboviral infections	16 (8.4)	6 (2.9)	22 (5.6)	
Participants who mentioned one to three symptoms of arboviral infections	142 (74.3)	63 (30.9)	205 (51.9)	
Participants who mentioned none	33 (17.3)	135 (66.2)	168 (42.5)	
Mention two diagnostic methods of arboviral infections				<0.001
Participants who mentioned one to two symptoms of arbovirus infections	160 (83.8)	65 (31.9)	225 (57.0)	
Participants who mentioned none	31 (16.3)	138 (67.6)	169 (42.8)	
Knowledge of HCWs about arboviral infection				<0.001
Good	64(33.5)	13(6.4)	77(19.5)	
Poor	127 (66.5)	191(93.6)	318(80.5)	

Note: †Chi-square test of association; FCT=Federal Capital Territory; HCW: healthcare worker

**Diagnostic practices for arboviral infections amongst healthcare workers in Lagos and the FCT:** over 90.0% of the HCWs in FCT (91.2%) compared to 68.6% (131) in Lagos, had no prior training on arboviral infection (P<0.001). Also, 88.3% (53) of the HCWs in Lagos reported that they had over three years of training on arboviral infection, compared to only 33.3% (6) of the HCWs in the FCT (P<0.001). Additionally, 33.5% (64) of the HCWs in Lagos previously suspected or diagnosed an arboviral infection, compared to only 5.4% (11) in the FCT (P<0.001). In Lagos, 75.0% (48) of the HCWs diagnosed arboviral infection using laboratory testing compared 63.6% (7) in the FCT (P<0.001). Amongst HCWs that reported diagnosing arboviral infection using laboratory testing, all in Lagos (48) reported using polymerase chain reaction, compared to 57.1% (4) in the FCT (P<0.001). Overall, 33.5% (64) of the HCWs in Lagos had good knowledge about arboviral infection, compared to only 6.4% (13) of their counterpart in the FCT (P<0.001) (Table 3).

**Table 3:** diagnosis of arboviral infections by healthcare workers in Lagos and the FCT

Variables	Lagos (%)	FCT (%)	Total (%)	p value†
**Received training on arboviral diagnosis**				<0.001
Yes	60 (31.4)	18 (88.2)	78 (19.7)	
No	131 (68.6)	186 (91.2)	317 (80.3)	
**Year of training (N=55)**				<0.001
Less than 1 year	0 (0.0)	5 (27.8)	5 (6.4)	
2 to 3 years	7 (11.7)	7 (38.9)	14 (17.9)	
3 and above	53 (88.3)	6 (33.3)	59 (75.6)	
**Ever suspected or diagnose arbovirus infections**				<0.001
Yes	64 (33.5)	11 (5.4)	75 (19.0)	
No	127 (66.5)	193 (9.5)	320 (81.0)	
**Diagnostic approach (N=75)**				<0.001
Clinical diagnosis	16 (25.0)	4 (36.3)	20 (26.7)	
Laboratory testing	48 (75.0)	7 (63.6)	55 (73.3)	
**Lab arboviral diagnostic method (N=55)**				<0.001
Rapid test and ELISA	0 (0.0)	3 (42.9)	3 (5.5)	
PCR	48 (100)	4 (57.1)	52 (94.5)	

Note: †Chi-square test of association; FCT=Federal Capital Territory; HCW: healthcare worker; N=number; PCR= polymerase chain reaction; ELISA= enzyme linked immunosorbent assay

Bivariate analysis of sociodemographic characteristics associated with diagnosis of arboviral Infections among healthcare workers: in Lagos State, sex (P: 002), age (P<0.001), cadre (P<0.001), knowledge of arboviral infection (P: 014) and prior training on arboviral diagnosis (P<0.001) were significantly associated with previous diagnosis of arboviral infections amongst HCWs attained 20% significance and were incorporated into the multivariate logistic regression analysis (p<0.20). Facility level of HCWs (P: 174) was found to be associated with previous diagnosis of arboviral infection at 20% significance level. However, only prior training on arboviral diagnosis was significantly associated with previous diagnosis of arboviral infection amongst HCWs in the FCT (P: 006). Also, knowledge about arboviral infection and diagnosis (P: 19) was associated with previous diagnosis of arboviral infections amongst at 20% significance level (Table 4).

**Table 4:** bivariate analysis of factors associated with the diagnosis of arboviral infections by healthcare workers in Lagos and the FCT

Ever diagnose Arboviral infection in your health facility						
**Variables**	**Lagos**			**FCT-Abuja**		
	Yes	No	p value†	Yes	No	p value†
Sex						
Male	38 (59.3)	46 (37.1)	0.002*	4 (40.0)	70 (36.1)	0.522
Female	26 (40.6)	81 (65.3)		6 (60.0)	124 (63.9)	
Age						
Less than 24	8 (12.5)	49 (38.6)	<0.001*	0 (0.0)	25 (12.9)	0.417*
25 to 34	8 (12.5)	32 (25.2)		5 (50.0)	71 (36.6)	
35 and above	48 (75.0)	46 (36.2)		5 (50.0)	98 (50.5)	
Profession						
Medical doctors	31 (48.4)	32 (25.1)	<0.001*	3 (30.0)	49 (25.3)	
Nurses	6 (9.3)	45 (35.4)		2 (20.0)	81 (41.8)	0.367
Lab scientist	27 (42.2)	50 (39.3)		5 (50.0)	64 (33.0)	
Facility level						
Secondary	56 (87.5)	101 (79.5)	0.174*	10 (100)	194 (100)	-
Tertiary	8 (12.5)	26 (20.5)		0 (0.0)	0 (0.0)	
Year of experience						
Less than 5	11 (17.2)	31 (24.6)	0.505	4 (40.0)	75 (38.7)	0.996
6 to 10 years	18 (28.1)	33 (26.2)		3 (30.0)	60 (30.9)	
More than 10 years	35 (54.7)	62 (49.2)		3 (30.0)	59 (30.4)	
Knowledge of arboviral infection and diagnosis						
Good	2 (9.5)	62 (36.5)	0.014*	3 (30.0)	10 (5.2)	0.19*
Poor	19 (90.5)	108 (63.5)		7 (70.0)	184 (94.8)	
Received training on arboviral diagnosis						
Yes	51 (79.7)	9 (7.1)	<0.001*	6 (60.0)	14 (7.2)	0.006*
No	13 (20.3)	118 (92.9)		4 (40.0)	180 (92.8)	

Note: †Chi-square test of association; *Fisher’s exact test; FCT= Federal Capital Territory

Multivariate analysis of the level of association with previous diagnosis of arboviral infection amongst healthcare workers in Lagos and the FCT: in Lagos State, male HCWs were 2.5 times more likely to have previously diagnosed arboviral infection (AOR=2.5, 95%CI: 1.2-4.8, p=0.003). Also, HCWs aged ≥35 years were more likely than those <24 years to have previously diagnosed arboviral infections (AOR=6.4, 95%CI: 2.7-14.9, P<0.001). Medical doctors (AOR=7.3, 95%CI: 2.7-19.5, P<0.001) and laboratory scientist (AOR=4.1, 95%CI: 1.5-10.7, P: 0.005) were more likely than nurses to have previously diagnosed arboviral infection. Also, HCWs with good knowledge (AOR=5.5, 95%CI: 1.2-24.2, P: 0.003) and have received training on arboviral diagnosis (AOR=51.4, 95%CI: 20.7-127.9, P<0.001) were more likely to have previously diagnosed arboviral infection (Table 5). In the FCT, HCWs with good knowledge about arboviral infection and diagnosis was 7.9 times more likely to diagnose an arboviral infection (AOR=7.9, 95%CI: 1.8035.2, P: 0.004). Similarly, HCWs that had previously received training on arboviral diagnosis were 8.6 times more likely to have diagnosed an arboviral infection (AOR=8.6, 95%CI: 2.2-33.9, P: 02) (Table 5).

**Table 5:** logistic regression of factors associated with the diagnosis of arboviral infection by healthcare workers in Lagos and the FCT

	Ever diagnosed Arboviral Infections			
**Variables**	**Lagos**		**FCT-Abuja**	
	Odds ratio (95% CI)	p-value	Odds Ratio (95% CI)	p-value
Sex				
Female	R			
Male	2.5(1.2 - 4.8)	0.003*	1.2 (0.3 - 4.3)	0.802
Age				
Less than 24	R			
25 to 34	1.5 (0.5 - 4.5)	0.438	-	-
35 and above	6.4 (2.7 - 14.9)	<0.001*	1.4 (0.4 - 4.9)	0.621
Profession				
Nurses	R			
Medical doctor	7.3 (2.7 - 19.5)	<0.001*	2.5 (0.4 - 15.4)	0.329
Lab scientist	4.1 (1.5 - 10.7)	0.005*	3.2 (0.5 - 16.8)	0.177
Knowledge of arboviral infection and diagnosis				
Poor	R			
Good	5.5 (1.2 - 24.2)	0.003*	7.9 (1.8 - 35.2)	0.004*
Received training on arboviral diagnosis				
No	R			
Yes	51.4 (20.7 -127.9)	<0.001*	8.6 (2.2 - 33.9)	0.02*

OR-odds ratio, CI-confidence interval, R-reference, FCT-Federal Capital Territory

## Discussion

The study was conducted to provide adequate information on the practices of HCWs toward the diagnosis of arboviral infections in Lagos and FCT-Abuja, Nigeria. The findings revealed several disparities between the two States, which are crucial to understanding key challenges with arbovirus surveillance and informing targeted interventions. This study revealed disparities in knowledge levels and diagnostic practices in the two regions. Generally, <20% of the HCWs had good knowledge about arboviral infection and diagnosis. While this is poor, our study found a higher proportion of HCWs with good knowledge compared to another study conducted in the Republic of Guinea where only 1% of the HCWs had good knowledge about arboviral infection [25]. The disparity in this finding could be a result of the differences in tools used to assess knowledge and the variation in the cut-offs set for good knowledge. However, over 80% of the HCWs had poor knowledge about arboviral infection, and, poor knowledge of arboviral infection could greatly affect the timely surveillance and response to arboviral infection outbreaks, therefore putting Nigerians at risk of adverse health outcomes. Our study found that more of the HCWs in Lagos exhibited good knowledge about arboviral infections compared to those in the FCT. While there are no studies in Nigeria that have assessed State-level disparities in knowledge about arboviral infections, a possible reason for this disparity can be a result of the higher proportion of HCWs in Lagos that were trained on arboviral infection compared to the FCT as identified in our study. This has been supported by a systematic review of studies, which found that training was effective in improving knowledge about arboviral infections [26]. Also, training on arboviral infection among HCWs in Lagos and the FCT was found to be associated with increased odds of previously suspecting and diagnosing arboviral infection. This could explain why good knowledge influenced the suspicion and diagnosis of the disease, as it is expected that training will improve knowledge and good knowledge will also increase the consciousness of HCWs to identify possible symptoms of arboviral infection.

In our study, male HCWs were more likely to suspect and diagnose an arboviral infection. This is similar to a study that found the male gender to be more likely to have moderate practices towards dengue fever prevention in Ethiopia [27]. The majority of the male HCWs were doctors, and their involvement in consultation with patients makes them more likely to suspect and clinically diagnose cases of arboviral infections. Although insignificant in the FCT, our study also found older age to be associated with increased odds to suspect and detect arboviral infection among HCWs in Lagos. In a study conducted to assess the knowledge and practice of HCWs towards COVID-19 in Peru, a similar finding was reported, as older HCWs were found to be more likely to have good practices towards COVID-19 [28]. This is suggestive that older HCWs may have more years of experience and are more likely to be knowledgeable about the clinical presentation of arboviral infections and diagnose these diseases.

In our study, laboratory scientists and medical doctors in Lagos State were found to have higher odds of diagnosing an arboviral infection. This is similar to a study conducted in eastern Ethiopia which found that physicians were more likely to have good practices towards Dengue disease prevention compared to nurses [27]. A possible reason for this could be the higher level of capacity of the medical doctors to detect the potential of the disease based on clinical presentation and their tendency to refer suspected cases to the laboratory for further testing, whereas, nurses are only involved in care rather than the detection of the disease. Generally, factors influencing diagnosis rates varied between the two States. Compared with their female counterparts, male HCWs in Lagos exhibited a significantly greater likelihood of diagnosing arbovirus infections, whereas sex did not significantly influence HCW's diagnosis of arboviral infections in the FCT.

**Limitations and strengths of the study:** the study has the following limitations. Firstly, there may be an information bias given some aspects of the collected data were self-reported. In addition, social desirability bias might have occurred in some instances because respondents may have responded to interview questions in a way that they best believe is socially acceptable rather than being completely accurate. However, probing questions were asked to ensure correct responses were possible. Secondly, our study was cross-sectional design, and thus we are unable to estimate changes in knowledge and arboviral diagnostic practice over time, making the relationship between variables and arboviral diagnostic practice not causal. However, the study can provide a baseline for future studies and provide evidence for government health agencies to carry out effective interventions to improve arboviral diagnostic practices among healthcare providers in Nigeria. By building upon existing knowledge and identifying region-specific challenges, this study contributes to a deeper understanding of the complexities involved in diagnosing arboviral infections and informs targeted strategies for improving arbovirus surveillance in Nigeria.

## Conclusion

Our study highlights significant disparities in HCWs´ diagnostic practices for arboviral infections between Lagos and FCT-Abuja, Nigeria. Variations in age distribution, professional backgrounds, knowledge levels, and training significantly influence diagnostic approaches. While Lagos demonstrates higher prior knowledge and diagnostic rates, the FCT shows a preference for laboratory testing. Gender and age also impact diagnosis rates, with male workers and older age groups exhibiting greater likelihood. Strengthening training programs and promoting knowledge dissemination are vital for improving arbovirus diagnosis and surveillance across Nigerian States. Further research should explore interventions to address identified disparities and enhance public health response.

### What is known about this topic

- Arboviral infections are group of diseases that have potential for disease outbreak in endemic regions;
- Good knowledge about arboviral infections amongst HCWs can improve surveillance and response to arboviral disease outbreak.

### What this study adds

- This study reveals that there are regional disparities in the level of knowledge about arboviral infections amongst HCWs;
- The study also shows that adequate training and good knowledge about arboviral infection increases the likelihood of HCWs to diagnose arboviral infection.
